# Out of Mind, Out of Sight: Language Affects Perceptual Vividness in Memory

**DOI:** 10.1371/journal.pone.0036154

**Published:** 2012-04-30

**Authors:** Lisa Vandeberg, Anita Eerland, Rolf A. Zwaan

**Affiliations:** Department of Psychology, Erasmus University Rotterdam, Rotterdam, The Netherlands; University of Leicester, United Kingdom

## Abstract

We examined whether language affects the strength of a visual representation in memory. Participants studied a picture, read a story about the depicted object, and then selected out of two pictures the one whose transparency level most resembled that of the previously presented picture. The stories contained two linguistic manipulations that have been demonstrated to affect concept availability in memory, i.e., object presence and goal-relevance. The results show that described absence of an object caused people to select the most transparent picture more often than described presence of the object. This effect was not moderated by goal-relevance, suggesting that our paradigm tapped into the perceptual quality of representations rather than, for example, their linguistic availability. We discuss the implications of these findings within a framework of grounded cognition.

## Introduction

When we comprehend language, we create a mental representation of the situation that is described by the text [1], [2], [3]. Embodied theories of language propose that the information in such situation models is not merely abstract or symbolic, but rather is grounded in our bodily experiences [4], [5], [6]. This proposal implies that the representations we create or retrieve during language processing are similar to the representations we created when we actually experienced the respective situation. As a result, these conceptual representations include perceptual, lexical, semantic, and functional features and are shaped by people's world knowledge and beliefs [7]. There is ample evidence that people retrieve perceptual [8]–[10] and motoric [11], [12] information from memory while processing language. For example, they represent the visual features of a described object or action (such as shape, orientation, color, size, or direction of motion) while reading about a situation [13]–[21].

We ask whether language can affect the activation of a concept to such an extent that it improves or reduces the quality of a visual representation. Only a few studies that we know of have explored the quality of visual mental representations by manipulating the resolution [22], realism [23], vividness [24] or spatial frequency [15] of visual stimuli. In the current study we refer to a representation's quality in terms of *perceptual vividness*. We isolate the perceptual component of a representation in our definition of vividness, however, perceptual information is not exhaustive of a conceptual representation [10]. We make the critical assumption that when the perceptual component of a representation is activated to a greater extent, the availability of visual information -such as outline or color- will increase and thereby also what we call the vividness of a mental representation in memory. We assessed whether (a) the general activation of a concept, or (b) merely the activation of a concept's visual component affects vividness of a mental representation.

Many types of linguistic information can have an impact on the availability of concept information in memory. For example, objects that are present [25], visible [9], or spatially close [26] to a protagonist in a described situation are more accessible than objects that are absent, occluded, or farther away, respectively. For example, in one study participants read short stories describing a characters' view of an object that was either blocked (e.g., by a curtain) or not [9]. They were slower to respond to verification questions about objects that were occluded from view than to visible objects, suggesting that the accessibility of objects is reduced during recall when these objects are absent from the protagonist's view in a described situation. Another factor that affects the accessibility of an object is whether that object is goal-relevant. Information that is relevant to the protagonist's goal is retrieved faster than irrelevant information [27]–[32]. Responses to probe words are faster after reading texts in which a goal was achieved, compared to control texts that describe a simple completed action [27], [28]. This suggests that goal category information is more accessible than information that is irrelevant (neutral) to the goal.

To investigate whether and how concept availability affects the perceptual vividness of the associated conceptual representation in memory, we created short stories in which we manipulated both object presence (versus absence) and goal-relevance (versus irrelevance). In these described situations, object presence alters the visual components of a conceptual representation, whereas goal-relevance may alter the conceptual representation in a non-perceptual way. We formulated two main hypotheses.

The first major hypothesis is that increased availability of a concept in memory leads to increased perceptual vividness of the accompanying representation. Thus, both object presence and goal-relevance should exert an effect on recalled vividness (hypothesis 1). The most straightforward evidence would come from a pattern in which both object presence and goal-relevance increase perceptual vividness of the concept in memory. However, it is also possible that object presence and goal-relevance interact in specific ways. Out of the space of possible hypotheses in support of a mapping between concept availability and vividness, the following two seem particularly relevant.

Research on goal-relevance has shown a greater availability for objects that are relevant to the protagonist's goals rather than irrelevant [27]–[32]. As a result, presence or absence of an object may only be sufficiently salient for relevant objects. In other words, information about an object's presence may differentiate the availability of relevant concepts but not of irrelevant concepts [28]. In this case, we would expect an interaction in which an increased vividness for present compared to absent objects would only be detected for relevant objects (hypothesis 1a).

However, there is a parallel between our manipulation of presence and manipulations of negation in the literature. There is evidence that negated concepts do not simply reduce accessibility of a concept, but are even replaced by alternative opposites later in processing [33]–[36]. These robust effects of negation may result in a floor level accessibility to absent concepts, regardless of their goal relevance. In this case, we would expect an interaction in which an increased vividness for relevant compared to irrelevant objects would only be detected for present objects (hypothesis 1b).

The major alternative hypothesis is that only object presence affects perceptual vividness. Describing that an object is or is not present involves the visual aspects of a situation, whereas describing that an object is or is not relevant to a protagonist's goals does not. After all, if objects are present they are visible regardless of whether they are relevant to a protagonist's goals. Thus, this hypothesis predicts a main effect of presence but no main effect of goal relevance and no interaction (hypothesis 2).

## Experiment 1

### Method

#### Ethics Statement

All participants were recruited online and voluntarily subscribed for participation in all of the described experiments. Written consent was not obtained because the experiment was noninvasive. This is in accordance with departmental practice approved by the Ethics Committee of Psychology (ECP) at the Erasmus University Rotterdam, the Netherlands.

#### Participants

242 participants were recruited online through Amazon's Mechanical Turk (http://www.mturk.com). The sample had a mean age of 35 (SD = 12) and contained 153 females (63%). 225 participants (93%) reported being a native speaker of English. These demographics are based on 241 participants, because the demographic data of one participant were missing. This participant and the 16 participants that did not report English as their native language were not excluded from the sample, because inclusion did not alter the result pattern. All participants were residents of the USA and were compensated with $1.50 for their participation, which required approximately 25 minutes.

#### Materials and design

Two versions of twenty critical short stories were created (see Table S1). Each story consisted of five sentences. The first three sentences of each story introduced an event and included information about the characters, their actions, and a location (see example story 1). The fourth sentence introduced a critical object that was either absent or present in the described situation, which was defined by placing the articles “a” or “no” before the object noun. The object was relevant to the protagonist's goal in one version of the story (version a) but irrelevant to the protagonist's goal in the other version (b). Goal-relevance was foregrounded in the first three sentences; the fourth sentence was similar for both versions of the stories. The fifth and final sentence did not mention the object and was neutral with respect to the presence or goal-relevance of the object.
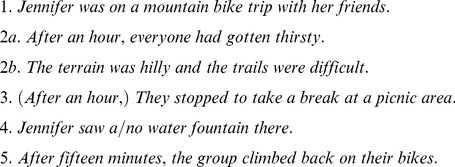
(1) Each story was paired with a picture that corresponded to the described object. Additionally, 20 filler picture-story pairs were created in which the picture did not correspond to the described object. The transparency level of all 40 pictures was adjusted to three different values using Adobe® Photoshop® software. A 50% transparency level served as a baseline condition, whereas a 45% transparency level reflected a slightly more transparent (less vivid) version of the picture and a 60% transparency level was slightly more opaque (more vivid), see Table S2. We based this asymmetry in transparency levels (45%, 50%, 60%) on a pilot study with identical absolute differences between pictures (40%, 50%, 60%). In this pilot study, people showed a tendency to select more opaque pictures over more transparent pictures when matching them to baseline pictures. In an attempt to select more comparable relative differences in transparency, we created a smaller absolute difference for the more transparent pictures than for the more opaque pictures.

This approach of mapping visual transparency levels onto representational vividness is inspired by work in social psychology [24]. In that study, people set the recalled transparency of previously processed pictures of a hot desert to less transparent (more opaque) when seated in a hot room than when seated in a cold room. The researchers interpreted this finding as evidence for perceptual fluency, in the sense that participants who experienced the visceral state of warmth, constructed more vivid and fluent mental representations of hot (versus cold) images. In the current study, we assessed how both *linguistic* factors affected recall of the transparency level of previously presented pictures.

We created two lists, one for goal-relevant and one for goal-irrelevant stories, thereby using goal-relevance as a between-subjects factor. In each list, half of the stories described the presence of an object and the other half described the absence of an object, thereby using object presence as a within-subjects factor. The lists were counterbalanced across subjects and the picture-story pairs were presented in randomized order within subjects.

#### Procedure

The experiment was programmed and presented in the Qualtrics survey research suite (http://www.qualtrics.com). Participants were instructed to (1) look at the presented picture, (2) read a short story, (3) decide which of two presented pictures best matched the picture they had seen previously, and (4) answer a question about the story. Each trial started with the presentation of a single picture. Critical pictures were presented at a 50% transparency level, whereas half of the filler pictures were presented at 45% and the other half at 60%. When participants clicked on a button on the screen, the picture disappeared and the story appeared. After reading the story and clicking a button, the 45% version of the previously seen picture appeared on the left of the screen and the 60% version appeared on the right. Participants indicated which version best matched the picture they had seen in the first part of the trial by checking the corresponding box. Additionally, a comprehension question that required a yes/no response followed one fourth of the trials to make sure that participants read the stories properly. After participants had answered the question, the next trial started. After completing all 40 trials, participants answered 20 questions about the absence or presence of the critical objects by checking the “yes” or “no” response box (e.g., “In the story about the mountain bike trip, did Jennifer see a water fountain in the picnic area?”). Finally, participants filled out several demographical questions.

### Results

**Figure 1:**
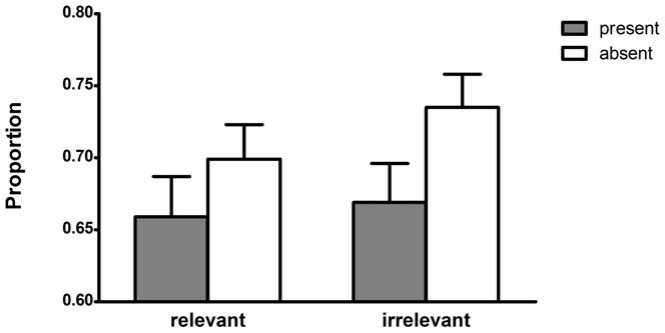
Proportion of hits on the most transparent picture per category in Experiment 1. Object presence represents a within-subjects factor and goal-relevance represents a between-subjects factor. Error bars represent the standard errors of the means.

Accuracy on the comprehension questions (M = 0.85; SD = 0.14) and the final questions about presence of the objects (M = 0.84; SD = 0.13) was high and above chance level (*t* (241) = 38.79, *p*<.0001 and *t* (241) = 39.14, *p*<.0001, respectively), indicating that participants properly read the stories. We calculated the average hit rate for both transparency levels (45% or 60%) across items for each participant, see Figure 1. Because the two hit rates are complementary, we only discuss the hit rates to the most transparent picture. In the following analysis and the analyses of Experiments 2 and 3, list was included as a between-subjects factor. Effects for the list variable are not reported, given the lack of theoretical relevance [37]. A 2 (presence)×2 (goal-relevance)×2 (list) mixed design ANOVA revealed only a significant main effect of presence (F (1,238) = 10.69, *p* = .001, ŋ^2^ = .043). Participants selected the most transparent picture more often when reading about an absent object (in 71.7% of the trials) than when reading about a present object (in 66.4% of the trials). Importantly, no other effects were significant, showing that goal-relevance did not significantly affect participants' decisions (main effect of goal-relevance: F (1,238) = 0.50, *p* = .48, ŋ^2^ = .00; interaction goal-relevance*presence: F (1,238) = 0.64, *p* = .43, ŋ^2^ = .00).

## Experiment 2

The results from Experiment 1 showed that participants selected the transparent picture more often when reading about an absent object than a present object. This suggests that the recalled vividness of the picture was reduced after reading about absence of the referent object in the described situation (relative to the object being present in the described situation). These findings demonstrate that vividness (quality) indeed is an aspect of perceptual representations, and that this aspect is affected by language. Furthermore, goal-relevance did not significantly affect participants' decisions. This finding supports our second main hypothesis that a change in availability of a concept in memory does not necessarily lead to an altered perceptual vividness of the accompanying representation. This suggests that the current paradigm isolates the perceptual quality of a concept rather than the overall concept availability in a more abstract way (the way a probe word might do). In order to (a) optimize the possibility of detecting an effect of goal-relevance by including it as a within-subjects factor, (b) assess whether participants were aware of the purpose of the experiment, and (c) replicate our findings, we performed a follow-up experiment.

### Method

#### Participants

229 participants were recruited online through Amazon's Mechanical Turk (http://www.mturk.com). This sample had a mean age of 32 (SD = 11) and contained 146 females (64%). 220 participants (96%) reported being a native speaker of English. The participants that did not report English as their native language were not excluded from the sample, because inclusion did not alter the result pattern. They were all residents of the USA and were compensated with $1.50 for their participation, which required approximately 25 minutes.

#### Materials and design

The materials and design were identical to those of Experiment 1, except that goal-relevance was treated as a within-subject factor. This resulted in 10 goal-relevant and 10 goal-irrelevant stories within a list, in half of which the object was present and half of which it was absent for each condition (5 relevant-present, 5 relevant-absent, 5 irrelevant-present, 5 irrelevant-absent). Four lists were created that were counterbalanced across subjects, the picture-story pairs were presented in randomized order within subjects.

#### Procedure

The procedure was identical to that of Experiment 1 except that participants were prompted about the purpose of the experiment. The prompt was presented after all the picture-story-picture trials but before the presence questions and the demographic questions. This prevented the participants from post-rationalizing the purpose of the study after focusing on absent versus present objects in the presence questions. They received the following instruction: “We would like to know what you think this survey is about. In the space below, please take your best guess at describing the purpose of this study”.

### Results

**Figure 2:**
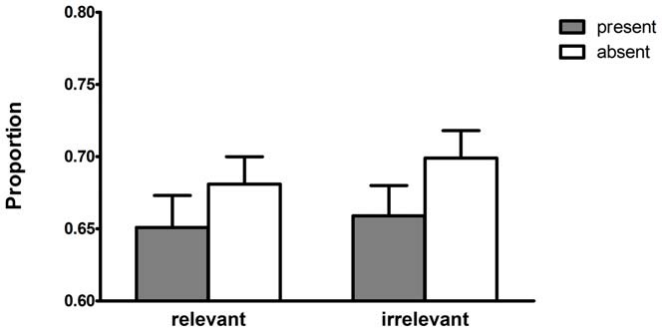
Proportion of hits on the most transparent picture per category in Experiment 2. Object presence and goal-relevance represent within-subjects factors. Error bars represent the standard errors of the means.

Accuracy on the comprehension questions (M = 0.85; SD = 0.15) and the final questions about presence of the objects (M = 0.83; SD = 0.14) was high and above chance level (*t* (225) = 36.02, *p*<.0001 and *t* (225) = 36.21, *p*<.0001, respectively), indicating that participants had properly read the stories. Four participants were excluded from the sample because, when prompted, they associated the presence of the objects in the stories with the selection of different transparency levels of the pictures. We calculated the average hit rate for each of both transparency levels across items for each participant (see Figure 2) and will only discuss the hit rates for the most transparent picture. Consistent with our findings from Experiment 1, a 2 (presence)×2 (goal-relevance)×4 (list) mixed design ANOVA revealed only a significant main effect of presence (F (1,221) = 5.42, *p* = .02, ŋ^2^ = .024)^3^; the transparent picture was selected in 69% of the trials with an absent object and 65.5% of the trials with a present object. Importantly, no other effects were significant, showing that goal-relevance did not affect participants' decisions (main effect of goal-relevance: F (1,221) = 1.14, *p* = .29, ŋ^2^ = .01; interaction goal-relevance*presence: F (1,221) = 0.22, *p* = .64, ŋ^2^ = .00).

## Experiment 3

The results of Experiment 2 demonstrate that transparency judgments were affected by presence of the target object in the referential situation, suggesting that object absence reduced the recalled vividness of an associated picture. Furthermore, the results again demonstrated that described goal-relevance did not affect transparency judgments. If the current transparency paradigm would provide a measure of concept availability per se, we would have expected goal-relevance to affect the results. Therefore, these findings suggest that our paradigm specifically taps into a perceptual aspect of conceptual representations.

A potential criticism to this conclusion could be that our manipulation of goal-relevance did not sufficiently distinguish between different levels of concept availability. If, for example, goal-relevance in our stories did not affect the availability of the stored concept, it would also not affect the perceived vividness of the concept. To ensure that our linguistic manipulations indeed resulted in distinct levels of concept availability (with a higher availability for present and relevant objects as opposed to absent and irrelevant objects), we performed a production experiment.

### Method

#### Participants

64 participants were recruited online through Amazon's Mechanical Turk (http://www.mturk.com). The sample had a mean age of 34 (SD = 13) and contained 42 females (66%). 60 participants (94%) reported being a native speaker of English. All participants were residents of the USA and were compensated with $0.60 for their participation, which required 10–13 minutes on average.

#### Materials and Procedure

The stories were identical to those in Experiment 2 but the final sentence was removed. No filler stories were included in this experiment, because the only aspect that distinguished fillers from experimental stories in the previous experiments was whether the presented pictures matched the object in the story or not. Because Experiment 3 did not include pictures, the filler stories no longer served a purpose. We created four lists with twenty stories (5 relevant-present, 5 relevant-absent, 5 irrelevant-present, 5 irrelevant-absent) that were counterbalanced across subjects and randomized within subjects. Again, the experiment was programmed and presented in the Qualtrics survey research suite. Participants were instructed to create the fifth missing sentence that fitted the preceding four given sentences from the story.

We aimed to measure the participants' accessibility to the object at the time of sentence creation by analyzing the content of the created sentences. The rationale behind this is that a greater semantic relatedness between the described object and the created sentence would demonstrate an enhanced availability of the concept. For this purpose, we computed for each condition the semantic overlap between the critical object (e.g., water fountain) and the produced sentences (e.g., “She went to the fountain and quenched her thirst.”) by means of Latent Semantic Analysis (LSA: http://lsa.colorado.edu/). LSA is a technique for computing the similarity of text pairs by comparing their vector representations in a multi-dimensional vector space, which is created from a large text corpus. Higher similarity values for stories in which the object was present or goal-relevant would demonstrate that present and goal-relevant stories indeed resulted in increased concept availability as compared to stories in the absent or irrelevant condition.

### Results

The similarity values computed by LSA ranged from −0.02 to 0.61. A 2 (object presence) * 2 (goal-relevance) * 4 (list)^3^ mixed design ANOVA revealed a significant main effect of presence (F (1, 60) = 111.06, *p*<.0001, ŋ^2^ = .65), showing that the produced sentences had greater semantic similarity to objects in the present rather than the absent condition (M = .29 and M = .16, respectively). Furthermore, a significant main effect of goal-relevance (F (1, 60) = 4.80, *p*<.05, ŋ^2^ = .07) revealed a greater semantic overlap between produced sentences and goal-relevant objects (M = .23) as compared to irrelevant objects (M = .21). Finally, analyses showed a significant two-way interaction of object presence and goal-relevance (F (1, 60) = 4.09, *p*<.05, ŋ^2^ = .06). Post-hoc paired-samples *t*-test revealed that the effect of goal-relevance only occurred for present objects (M_diff_present_ = 0.311−0.267 = 0.045, *t* (63) = 2.53, *p*<.05) but not for absent objects (M_diff_absent_ = 0.153−0.155 = −0.002, *t*<1). These results demonstrate that both the levels of object presence and goal-relevance as manipulated in the current study resulted in different degrees of concept availability.

## Discussion

We examined the quality of mental representations that are activated during language processing. Previous studies on perceptual representations manipulated visual object features to explicitly match or mismatch described object features. We did not. We manipulated the perceptual quality of the response options to the referent object and provided no matching decision alternative (both response options had a different transparency level than the original picture; thus, all answers were in principle incorrect). This manipulation revealed an implicit tendency of participants to select a more transparent (less vivid) picture after reading about a corresponding absent object. This implies that the entities that are not present in a described situation have a decreased perceivability in the referent representation. Studies that used probe words to assess the accessibility of visually absent, occluded, or distant objects found similar results in terms of availability of a corresponding concept [9], [25], [26]. Our current results extend these findings by specifying that a specific visual aspect of the concept representation is altered, namely its vividness.

To our knowledge, four previous studies have assessed perceptual quality [15], [22]–[24], only one of which is directly relevant to our research question [22]. Here, participants read sentences that manipulated the perceivability of an object (e.g., about a skier seeing a moose through fogged versus clean goggles), which facilitated response times to subsequently presented pictures of the object with a matching quality (low versus high visual resolution). Note that our current manipulation was more subtle. Our sentences did not mention perceived visual quality, they were embedded in stories in a task that did not require picture-to-sentence matching, and the pictures we presented in the experimental trials were not explicitly congruent or incongruent with the sentence in which the object was mentioned. Therefore, these findings are less susceptible to potential task-based strategies that participants might adopt to successfully perform the task. Any processing strategy in which participants did relate pictures to stories would have only been useful to distinguish experimental from filler stories, because this was the salient (but non-critical) manipulation.

Another difference is that we studied whether linguistic manipulations can qualitatively alter an experiential trace that was laid down within the experiment. All participants encoded the same pictures with identical (50%) transparency, thereby guaranteeing a baseline visual representation that was identical across conditions. Any difference in recollection of the encoded pictures should therefore be due to a difference in activation of this baseline trace. In other words, this paradigm taps into *re*-activation of a specific perceptual trace, rather than into a perceptual trace that might have been created ad hoc during language processing. For example, people may not have experience with seeing a moose through foggy goggles per se [22], but their (different) prior experience with the meaning of the words “foggy” and “moose” can still enable them to infer the approximate meaning of the sentence and envision a hazy moose. Therefore, it may be possible that people created a specific instant of a moose (of high or low visual quality), rather than recruiting a visual representation of a hazy moose from memory. For this reason, the current approach provides straightforward evidence that the *strengthening* of an experiential trace resulted in a *better quality* of the representation.

Whether a described object was relevant to the protagonist's goal did not affect the recalled transparency of encoded pictures. To ensure that this was not due to the stimuli we created, we performed a control experiment (Experiment 3) that demonstrated increased accessibility to relevant compared to irrelevant concepts, which confirms that our stimuli differentiated goal-relevance. A significant interaction revealed that the effect of relevance only occurred for stories in which the object was present. We hypothesized this pattern of concept availability based on the negation literature (hypothesis 1b) [33]–[36]. Overall, this pattern suggests that the concept availability of absent objects was reduced to floor level, resulting in an effect of goal-relevance in the production task only for present objects.

Even though our control experiment and previous research [27]–[32] convincingly showed that goal-relevance improves the accessibility of concept information, Experiments 1 and 2 did not reveal any effect of goal-relevance. This suggests that the current paradigm actually tapped into visual properties rather than overall concept availability or mere lexical accessibility, which supports hypothesis 2. It furthermore demonstrates that specific linguistic descriptions affect the mental representations we store in memory by differential reactivation of a perceptual trace. Based on these data alone, we cannot distinguish whether reading about absent objects did not recruit the stored perceptual information, or whether it did but the activation of the perceptual trace decayed more quickly. However, evidence from the negation literature suggests that early processing of negation/absence does not yet affect concept availability, only later processing does [33]–[36]. This suggests that the experiential trace of the referent concept might have been activated, but that it decayed (or was suppressed) shortly after. However, further research is needed to draw such conclusions within this framework.

People speak in terms of memories fading away, having a clear recollection of something, erasing images from one's mind, or having a situation fresh in memory. Our findings suggest that there is an actual physical component of mental representations that underlies these metaphors. In this study, we were able to isolate a qualitative visual component of concepts. This manifested itself in the recalled vividness of a stored representation. Even though objects were always explicitly mentioned in the text, the vividness of their corresponding representation in memory differed as a function of their described presence. These findings are in line with a grounded perspective on language processing, because they suggest that reading situation descriptions differentially re-activates experiential traces that were laid down previously.

The current approach provides new possibilities for future research. Given the wealth of demonstrations of perceptual involvement during language processing, we argue that at this point in time it is necessary to specify which aspects of concepts contribute to which aspects of language comprehension. This way, we will be able to formulate the strengths and weaknesses of the grounded (experiential) view on language processing and take new steps in understanding how people comprehend language and represent the external world.

## Supporting Information

Table S1 **Experimental stories.** Object presence is indicated by the articles “a” or “no” in sentence 4 of each story. Goal-relevance is presented in different columns: The left column contains stories in which the object is relevant; the right column contains stories in which the object is irrelevant.
(DOCX)

Table S2 **Experimental pictures with different transparency levels.** From left to right: 45%; 50%; 60%.
(DOCX)
