# Supplementary material for: Out of Mind, Out of Sight: Language Affects Perceptual Vividness in Memory
Source: PLoS One. 2012 Apr 30;7(4):e36154. doi: 10.1371/journal.pone.0036154 (PMC3340353; doi:10.1371/journal.pone.0036154)
Supplement: Table S1 — Experimental stories. Object presence is indicated by the articles “a” or “no” in sentence 4 of each story. Goal-relevance is presented in different columns: The left column contains stories in which the object is relevant; the right column contains stories in which the object is irrelevant. (DOCX) [file pone.0036154.s001.docx]

| **Object** | **Relevant** | **Irrelevant** |  |
| --- | --- | --- | --- |
| boat | Charlotte was stranded on a deserted island. | Charlotte was walking down the beach. |  |
|  | After a few days there was almost no food left. | It was early in the morning. |  |
|  | She desperately scanned the horizon | Both the beach and the ocean were remarkably quiet. |  |
|  | Charlotte saw a/no boat sailing by. | Charlotte saw a/no boat sailing by during her walk. |  |
|  | The sun was beating down. | Just as Charlotte wanted to go home, it started to rain. |  |
|  |  |  |  |
| bottle of water | Linda went out for lunch with her colleagues. | Linda went out for lunch with her colleagues. |  |
|  | They found a nice restaurant with a beautiful view. | They found a nice restaurant with a beautiful view. |  |
|  | When they sat down, Linda found herself very thirsty. | The waitress came to take their orders. |  |
|  | She saw that there was a/no bottle of water on the table. | Linda saw that there was a/no bottle of water on the table. |  |
|  | After Linda and het colleagues finished their lunch they went back to the office. | After they finished their lunch they went back to the office. |  |
|  |  |  |  |
| box | For his new job, Jimmy had to move to San Francisco. | To pay his rent, Jimmy worked night shifts in a factory. |  |
|  | He hired a moving company to move all his stuff. | After three hours of strenuous work, Jimmy had a break. |  |
|  | Half an hour after arrival of the company, Jimmy checked whether they started already. | Another worker took over for the time being. |  |
|  | When he looked into the truck, there was a/no box. | When he came back, there was a/no box on the conveyor belt. |  |
|  | An hour later he went to his landlord to hand in the keys. | Jimmy realized he had come back from his break too late. |  |
|  |  |  |  |
| chair | Today was Mel's first day on the job as a secretary. | Today, Mel started her new job as a secretary. |  |
|  | She was in the copy room to print some forms. | She started out by checking her email and making tea for her boss. |  |
|  | Suddenly, she felt dizzy. | Then she walked to the copying room to print some forms. |  |
|  | She noticed that there was a/no chair in the copy room. | She noticed that there was a/no chair in the copying room. |  |
|  | Later that day, she called the doctor for an appointment. | Then she realized that she forgot to bring her boss his tea and hurried back to the kitchen. |  |
|  |  |  |  |
|  |  |  |  |
|  |  |  |  |
| coffee machine | Fiona wanted to surprise her mom for Mother’s Day. | Fiona wanted to surprise her mom for Mother’s Day. |  |
|  | She entered the house using the key she kept and started cooking dinner. | She entered the house using the key she kept and started cooking a nice dinner. |  |
|  | She thought it would be nice to start with a hot beverage. | When the main course was almost done, she waited and looked around. |  |
|  | She saw a/no coffee machine in the kitchen. | She saw a/no coffee machine in the kitchen. |  |
|  | Then the timer sounded and she took the rice off the stove. | Then the timer sounded and she took the rice off the stove. |  |
|  |  |  |  |
| coin | Keith was working on his term paper in the computer room. | Keith was working on his term paper in the computer room. |  |
|  | After some time he craved a cigarette. | After some time he craved a cigarette. |  |
|  | On the way out, he walked to the vending machine in the hallway. | On the way out, he checked the vending machine in the corridor. |  |
|  | He found a/no coin in his wallet. | He found a/no coin left behind in the change slot. |  |
|  | Then he heard a familiar voice behind him. | Then he heard a familiar voice from behind him. |  |
|  |  |  |  |
| doormat | Peter was a plumber. | Peter was driving around in his delivery van. |  |
|  | He stopped in front of a big house for his first job of the day. | He had to deliver some packages before the end of his shift. |  |
|  | The sidewalk was very muddy. | He stopped in front of a big house. |  |
|  | As he walked up to the door, he saw a/no doormat. | As he walked up to the door, he saw a/no doormat. |  |
|  | He rang the doorbell. | Then suddenly, a dog started barking and Peter ran back to his van. |  |
|  |  |  |  |
| fence | Jake and Sally were hiking in the hills. | Jake and Sally were hiking in the hills. |  |
|  | They enjoyed the sunny weather as they came across a meadow with grazing cows. | They kept a swift pace and enjoyed the sunny weather. |  |
|  | Sally was afraid the cows would come too close. | After several hours, they came across a quaint little pasture. |  |
|  | There was a/no fence bordering the meadow. | There was a/no fence bordering it. |  |
|  | Just then, the clouds rolled in to cover the sun. | Just then, the clouds rolled in to cover the sun. |  |
|  |  |  |  |
|  |  |  |  |
|  |  |  |  |
|  |  |  |  |
|  |  |  |  |
| firewood | Josh and Mary had rented a vacation home. | Josh and Mary had rented a vacation home. |  |
|  | The house had a hot tub, wireless internet, and a fireplace. | It was situated beautifully at the top of a mountain. |  |
|  | It was cold at night. | The house had a hot tub, wireless internet, and a fireplace. |  |
|  | There was (no) firewood in the house. | There was (no) firewood in the house. |  |
|  | Josh and Mary opened up a bottle of red wine. | Josh and Mary enjoyed the hot tub. |  |
|  |  |  |  |
| magazine | Fred was waiting at the hairdresser's. | Fred was waiting at the hairdresser's. |  |
|  | He was the third person in line for a haircut. | He was the third person in line for a haircut. |  |
|  | Fred was bored and wanted something to read. | He looked around and observed the other customers. |  |
|  | There was a/no magazine on the table. | There was a/no magazine on the table. |  |
|  | A few minutes later, it was Fred's turn already. | A few minutes later, it was Fred's turn already. |  |
|  |  |  |  |
| peppermint | Annie was on a long hiking tour. | Annie was on a long skating tour. |  |
|  | After a few hours, she started to feel unwell. | After one hour she stopped to take a rest. |  |
|  | Her throat was aching. | Her legs hurt a little. |  |
|  | She had a/no peppermint in her pocket. | She had a/no peppermint in her pocket. |  |
|  | Then her mobile phone went off. | After ten minutes she started skating again. |  |
|  |  |  |  |
| piece of pie | Bob was invited to a birthday party on Friday evening. | Bob arrived at a birthday party on Friday evening. |  |
|  | He went to the party straight from work. | It had been a very busy week at work. |  |
|  | He did not have time for dinner so he was hungry. | He was tired and wanted to sit down. |  |
|  | At the party there was a/no piece of pie left for him. | There was a/no piece of pie left for him. |  |
|  | After a couple of hours, Bob went home and fell asleep quickly. | After about an hour, Bob went home and fell asleep quickly. |  |
|  |  |  |  |
| pyramid | Joan was on holiday in a small town in Egypt. | Joan was on holiday in a small town in Egypt. |  |
|  | She went diving almost every day. | She went diving almost every day. |  |
|  | But she was also dying to see some of the ancient Egyptian architecture. | In the evenings she went to her favorite restaurant. |  |
|  | In the small town there was a/no pyramid. | In the small town there was a/no pyramid. | |
|  | Joan had decided on coming back next year. | Joan had decided on coming back next year. |  |
|  |  |  |  |
| sea shell | Jenna planned on going to the beach, since the weather was lovely. | Jenna planned on going to the beach, since the weather was lovely. |  |
|  | She packed her bag and drove to the coast. | She packed her bag and drove to the coast. |  |
|  | Jenna liked collecting pretty things from the beach. | Once she got there, she decided to go for a swim in the ocean straight away. |  |
|  | When her feet touched the water, she looked down where she saw a/no sea shell. | When her feet touched the water, she looked down where she saw a/no sea shell. |  |
|  | The water wasn't as warm as she hoped and she walked back to her towel. | The water wasn't as warm as she hoped and she walked back to her towel. |  |
|  |  |  |  |
| slide | Milly took her little nephew to the zoo. | Milly took her little nephew to the zoo. |  |
|  | They ended up at a little playground. | He was scared of the lions, but loved the monkeys. |  |
|  | After a while Milly's nephew was tired of the swings and wanted to play somewhere else. | At the end of the day they ended up at a little playground. |  |
|  | Milly saw that there was a/no slide there. | Milly saw that there was a/no slide there. |  |
|  | After playing for a while Milly brought her nephew back home. | After playing for a while Milly brought her nephew back home. |  |
|  |  |  |  |
| stamp | Sam was about to go out for a walk. | Sam just woke up and was about to have breakfast. |  |
|  | His roommate asked Sam to post his letter. | He was hungry, so he decided to make some toast. |  |
|  | Sam grabbed the envelope to make sure the postal charges were covered. | Then he noticed an envelope on the table with his name on it. |  |
|  | There was a/no stamp in the upper right corner. | There was a/no stamp in the upper right corner. |  |
|  | Then he realized his friends would come over today. | Before he could open the envelope, a weird smell made him realize that his toast was burned. |  |
|  |  |  |  |
| tree | Will went to help his brother paint his new house. | Will went to help his brother paint his new house. |  |
|  | It was pretty warm that day. | It was old and needed a lot of work, but it was a cozy place. |  |
|  | At lunch they wanted to sit outside in the shadow. | When they had enough of it, they sat down and relaxed in the back yard. |  |
|  | There was a/no tree in the backyard. | There was a/no tree there. |  |
|  | Will offered to begin with the living room. | Will offered to come back the next day to finish the work. |  |
|  |  |  |  |
|  |  |  |  |
| water fountain | Jennifer was on a mountain bike trip with her friends. | Jennifer was on a mountain bike trip with her friends. |  |
|  | After an hour, everyone had gotten thirsty. | The terrain was hilly and the trails were difficult. |  |
|  | They stopped to take a break at a picnic area. | After an hour, they stopped to take a break at a picnic area. |  |
|  | Jennifer saw a/no water fountain there. | Jennifer saw a/no water fountain there. |  |
|  | After fifteen minutes, the group climbed back on their bikes. | After fifteen minutes, the group climbed back on their bikes. |  |
|  |  |  |  |
| weighing scale | Vanessa walked down the supermarket aisle. | Vanessa was doing some shopping. |  |
|  | She wanted to make a dessert with fruit. | She wanted to make a dessert for her friends. |  |
|  | The recipe required exactly 200 milligrams of black berries and cherries. | She walked to the storing shelf with fresh fruit. |  |
|  | She looked around and saw there was a/no weighing scale. | There was a/no weighing scale. |  |
|  | Ten minutes later she walked out of the supermarket. | Fifteen minutes later she walked out of the supermarket. |  |
|  |  |  |  |
| whiskey bottle | Bill landed at the airport late at night. | Bill landed at the airport late at night. |  |
|  | An hour later, he checked into his hotel room. | An hour later, he checked into his hotel room. |  |
|  | Bill felt like having a drink before going to sleep. | Bill decided to relax a little before going to sleep. |  |
|  | He saw that there was a/no whiskey bottle in the minibar. | He saw that there was a/no whiskey bottle in the minibar. |  |
|  | Then he remembered he promised to call his wife upon arrival. | Feeling quite exhausted after all, he went to the bathroom to brush his teeth. |  |
|  |  |  |  |
