# Supplementary material for: Out of Mind, Out of Sight: Language Affects Perceptual Vividness in Memory
Source: PLoS One. 2012 Apr 30;7(4):e36154. doi: 10.1371/journal.pone.0036154 (PMC3340353; doi:10.1371/journal.pone.0036154)
Supplement: Table S2 — Experimental pictures with different transparency levels. From left to right: 45%; 50%; 60%. (DOCX) [file pone.0036154.s002.docx]

|  |  | | |
| --- | --- | --- | --- |
| **Object** | **45%** | **50%** | **60%** |
| boat | 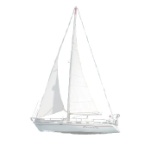 | 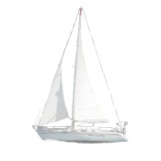 | 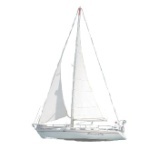 |
| bottle of water | 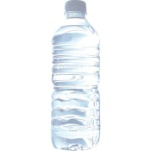 | 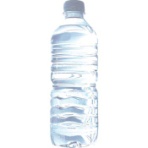 | 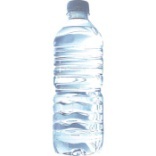 |
| box | 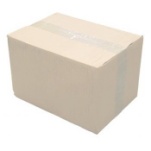 | 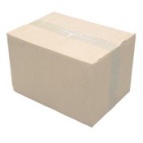 | 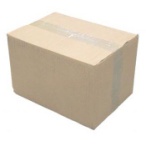 |
| chair | 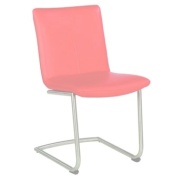 | 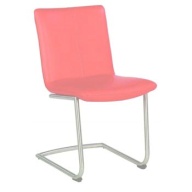 | 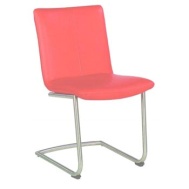 |
| coffee machine | 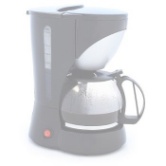 | 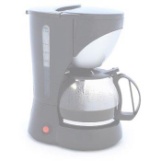 | 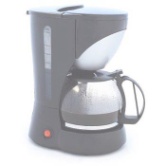 |
| coin | 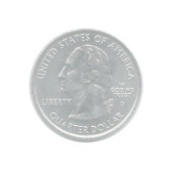 | 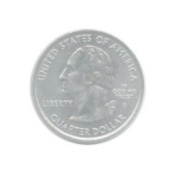 | 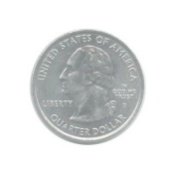 |
| doormat | 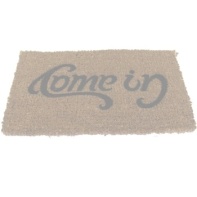 | 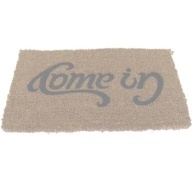 | 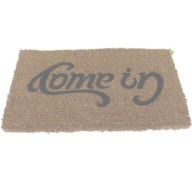 |
| fence | 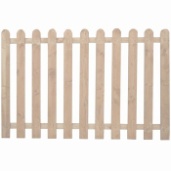 | 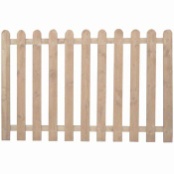 | 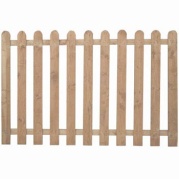 |
| firewood | 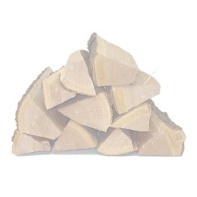 | 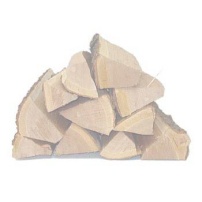 | 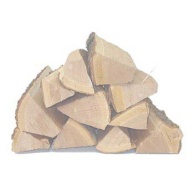 |
| magazine | 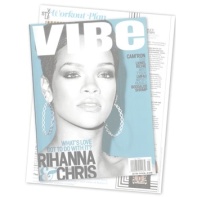 | 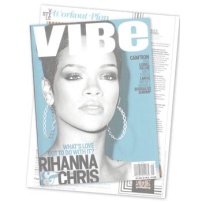 | 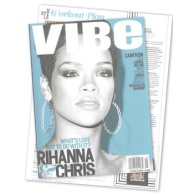 |
| peppermint | 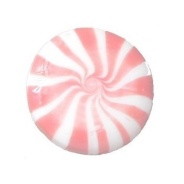 | 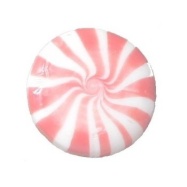 | 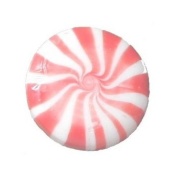 |
| piece of pie | 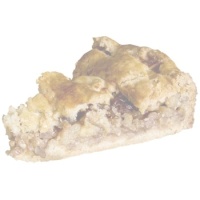 | 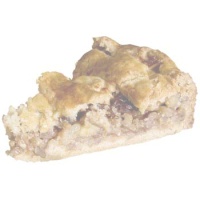 | 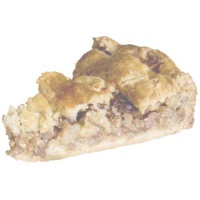 |
| pyramid | 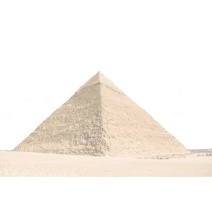 | 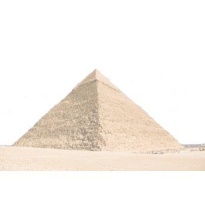 | 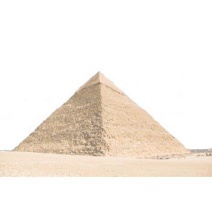 |
| sea shell | 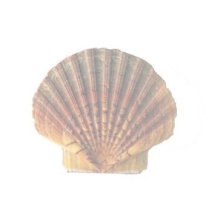 | 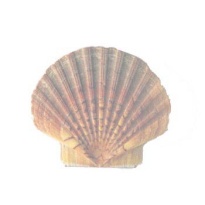 | 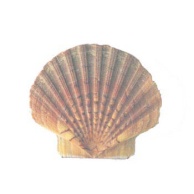 |
| slide | 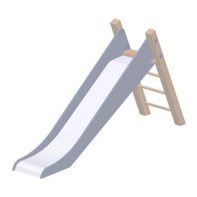 | 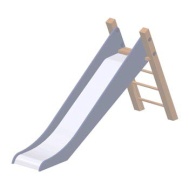 | 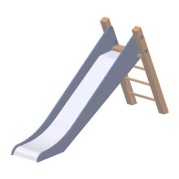 |
| stamp | 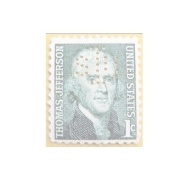 | 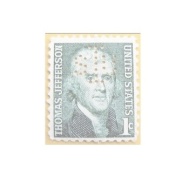 | 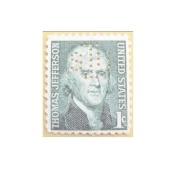 |
| tree | 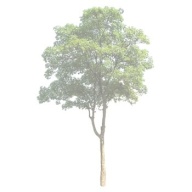 | 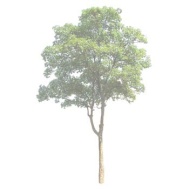 | 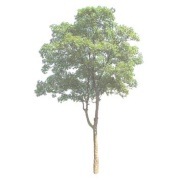 |
| water fountain | 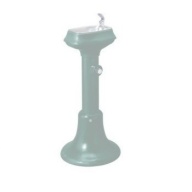 | 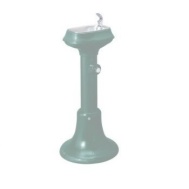 | 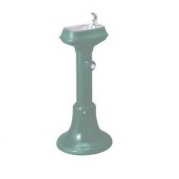 |
| weighing scale | 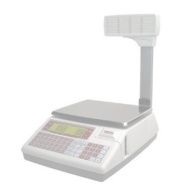 | 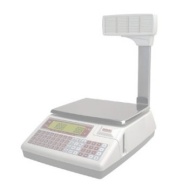 | 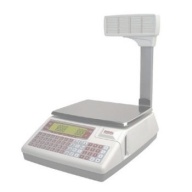 |
| whiskey bottle | 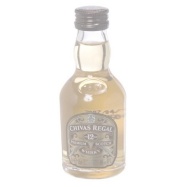 | 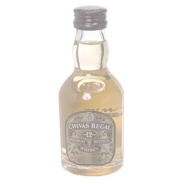 | 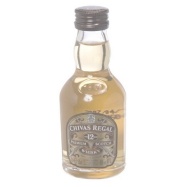 |
